# Supplementary figures and images for: A New Insight into MYC Action: Control of RNA Polymerase II Methylation and Transcription Termination
Source: Biomedicines. 2023 Jan 30;11(2):412. doi: 10.3390/biomedicines11020412 (PMC9952900; doi:10.3390/biomedicines11020412)

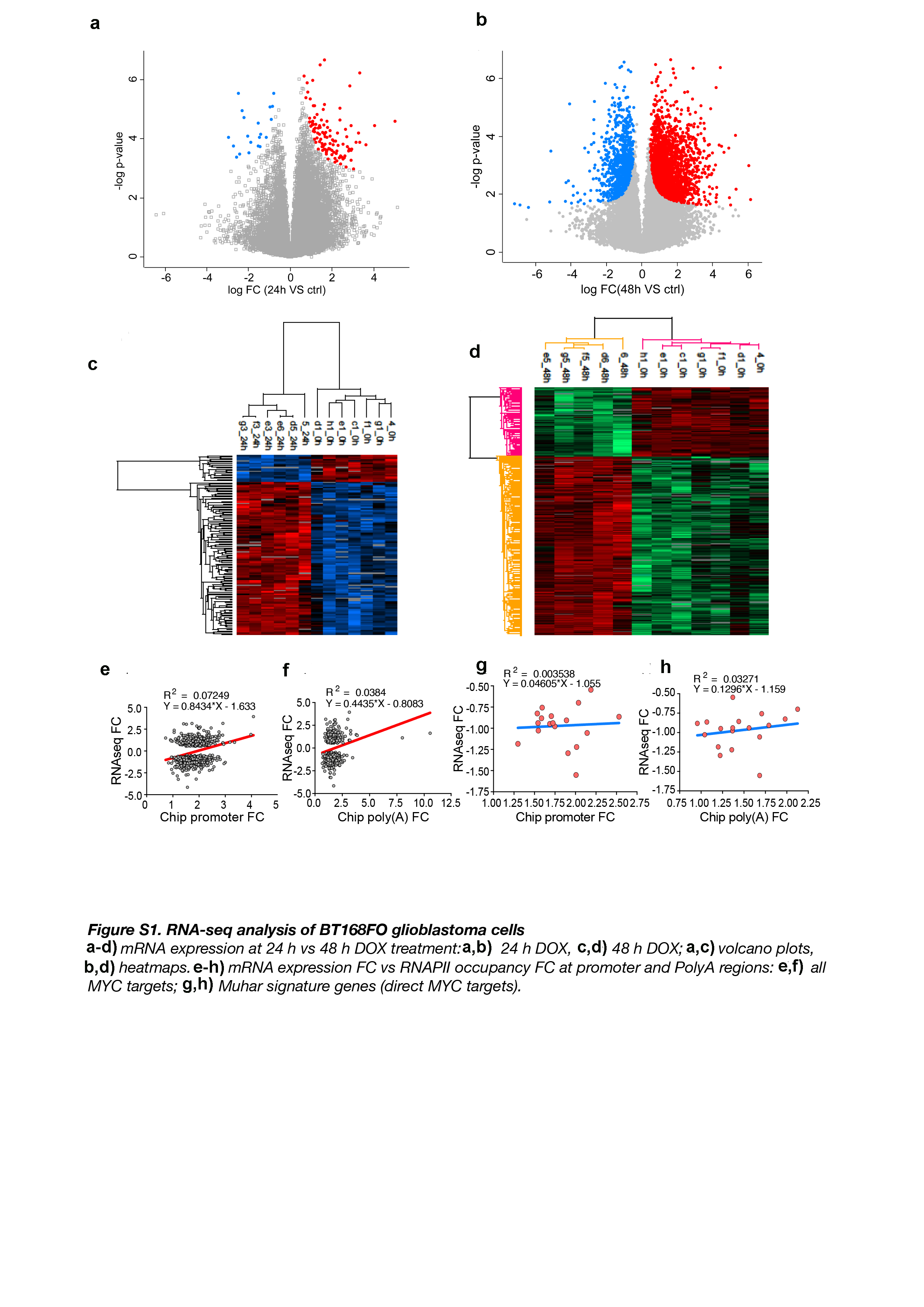

Supplement: Supplementary file 1 [file biomedicines-11-00412-s001.zip › Supplementary Figure S1.tif]
